# Computational methods for chromosome-scale haplotype reconstruction

**DOI:** 10.1186/s13059-021-02328-9

**Published:** 2021-04-12

**Authors:** Shilpa Garg

**Affiliations:** Department of Biology, University of Copenhagen, Copenhagen, Denmark

## Abstract

High-quality chromosome-scale haplotype sequences of diploid genomes, polyploid genomes, and metagenomes provide important insights into genetic variation associated with disease and biodiversity. However, whole-genome short read sequencing does not yield haplotype information spanning whole chromosomes directly. Computational assembly of shorter haplotype fragments is required for haplotype reconstruction, which can be challenging owing to limited fragment lengths and high haplotype and repeat variability across genomes. Recent advancements in long-read and chromosome-scale sequencing technologies, alongside computational innovations, are improving the reconstruction of haplotypes at the level of whole chromosomes. Here, we review recent and discuss methodological progress and perspectives in these areas.

**Supplementary Information:**

The online version contains supplementary material available at 10.1186/s13059-021-02328-9.

## Introduction

Haplotypes are combinations of alleles from multiple genetic loci on the same chromosome that are inherited together; the term haplotype can encompass as few as two loci or refer to a whole chromosome (that is, chromosome-scale haplotype). For diploid genomes, a given length of chromosomal DNA will have two haplotypes, one inherited from each parent, whereas several haplotypes exist for any given chromosomal region at the population level or for polyploid genomes. DNA microarrays and short-read sequencing can determine the collection of alleles at genetic loci (that is, genotypes) but provide no information at the level of haplotypes, whether alleles are co-located on the same copy of a chromosome, or which of the parental chromosomes harbors a particular allele. Hence, computational reconstruction of haplotypes using upcoming sequencing technologies, by either read mapping to a reference genome or de novo assembly, is required.

Haplotype information is fundamental for medical and population genetics [1, 2], where it is used to study genetic variation associated with human diseases [3, 4]. Traditionally, specific SNP locus-specific association to diseases was studied with respect to a linear reference sequence, for example, two SNPs, rs9494885 and rs2230926 in the TNFAIP3 gene w.r.t Grch37 reference, have known correlation with scleritis disease [5]. However, individual haplotypes (or their collection in the form of a pan-genome graph [6], which represents the genetic variations from populations and medical samples) can help to discover highly complex variations such as nested structural variation, inversions, and other complex rearrangements (reviewed in [7]) and to access the full spectrum of rare inherited variants and de novo mutations [8]. For example, the haplotype information is helpful to detect a rare case of keratitis-ichthyosis-deafness syndrome that exhibits a spontaneous correction of a pathogenic mutation by another mutation on the whole-chromosome scale [9]. Additionally, the phenomenon of compound heterozygosity on homologous chromosomes is responsible for recessive Mendelian disorders [4]. The chromosome-scale haplotypes also have functional relevance—the distribution of *cis*- and *trans*-acting variants between homologous chromosomes, that is, the phase of variants, can affect gene expression; chromosome-scale haplotypes help study interactions between variants in regulatory elements (long-range promoter-enhancer interactions) [4]. Another highly relevant chromosome-scale haplotyping example is to understand the context of aneuploidy (chromosome loss or gain) in cancer genomes, for example, large copy number gain in centromere 17 for chromosomal instability in breast cancer [10] also requires recent haplotyping approaches. The inference of whole-chromosome haplotypes has clinical relevance: having both variants on the same allele (cis) lead to a specific (for example, super-responder) phenotype, while those variants were on separate alleles (trans) do not. Haplotypes also play an important role in understanding the interplay of evolutionary processes that shape genetic variation, such as recombination, gene conversion, mutation, and selection. For example, modification of plant breeding strategies based on evolutionary processes identified through haplotype reconstruction can result in agricultural improvements [11]. Another highly relevant application occurs in the analysis of viral infections [12], where haplotype reconstruction can help to identify drug resistance and virulence factors and aid treatment decisions [13, 14].

Despite recent advances, sequencing technologies are limited in their ability to cover repetitive genomic regions to produce chromosome-scale haplotypes. Therefore, local (short-range) and genome-wide (long-range) information must be computationally integrated to assemble chromosome-scale haplotypes [15]. The integrative algorithms used for reconstruction must be tuned for the specific genome characteristics of a species, such as genome size, number of haplotypes, and repeat or haplotype variation. Many large-scale sequencing initiatives, such as the Vertebrate Genomes Project [16], the DNA Zoo project (https://www.dnazoo.org/), Darwin Tree of Life (https://www.darwintreeoflife.org/), the Human Microbiome Project (https://www.hmpdacc.org/), and the Human Pangenome Project (https://humanpangenome.org/), have begun to leverage diverse recent sequencing data types (Table 1) to reconstruct haplotypes for various species. These projects have designed and integrated bioinformatic pipelines in a common platform for large-scale genome analyses [24].

**Table 1:** Third-generation sequencing initiatives and reference data sets

Initiatives	# samples/#haplotypes	Technologies	Links
Genome in a Bottle [17, 18] (GIAB)	2 trios and 1 sample, 6 haplotypes	PacBio, ONT, Illumina, BioNano, Strand-seq, 10xG	ftp://ftp-trace.ncbi.nlm.nih.gov/ReferenceSamples/giab/data/
Human Genome Structural Variation Consortium [15] (HGSVC)	> 3 trios, > 6 haplotypes	PacBio, Illumina, BioNano, Hi-C, Strand-seq, 10xG	https://www.internationalgenome.org/data
Vertebrate Genome Project (VGP; facilitated by Genome 10 K), Darwin Tree of Life Project	> 100, ongoing haplotyping efforts	10xG, PacBio, Hi-C	https://vgp.github.io/genomeark/
Human Pangenome Project	> 10, > 20 haplotypes	PacBio, ONT, Hi-C	https://s3-us-west-2.amazonaws.com/human-pangenomics/index.html?prefix=HPRC/
Earth Biogenome Project (facilitated by Genome 10 K)	> 10, ongoing haplotyping efforts	PacBio, Hi-C	https://www.earthbiogenome.org/publications
The DNA Zoo project	> 10, ongoing haplotyping efforts	Hi-C and WGS	https://www.dnazoo.org/
Japanese Reference Project [19] (1KJPN)	> 1, > 2 haplotypes	PacBio, Illumina	https://jrg.megabank.tohoku.ac.jp/en
CHM1, CHM13 [20], HX1 [21], PGP-1 [22], AK1 [23]	Individual samples, two haplotypes each (except CHM1 and CHM13)	PacBio, ONT, BioNano, Hi-C, Illumina	n/a

In this Review, we discuss the bioinformatic methods—reference-based de novo and strain-resolved metagenome assembly—to reconstruct haplotypes in diploids, polyploids, and microbial communities. We present the strengths and weaknesses of these methods, alongside examples of their biological applications. Finally, we conclude with challenges and future directions, with an emphasis on both the algorithmic and technological advances required to achieve routine high-quality haplotypes for further biological discoveries.

## Evolution of sequencing technologies

Early advancements in sequencing technologies [25], such as next-generation sequencing with read lengths of 150–250 bp and accuracy > 99.8%, revolutionized haplotype reconstruction [26–28] and helped to characterize the genomic landscape. However, the fairly short read lengths limit the ability to uniquely span repeats and identify regions of heterozygosity, and these technologies are unable to produce whole-chromosome haplotypes. More recently, developments in long-read sequencing technologies [25, 29] have begun to substantially increase the utility and application of haplotype reconstruction.

### Short-range sequence and haplotype information

**Fig. 1:**
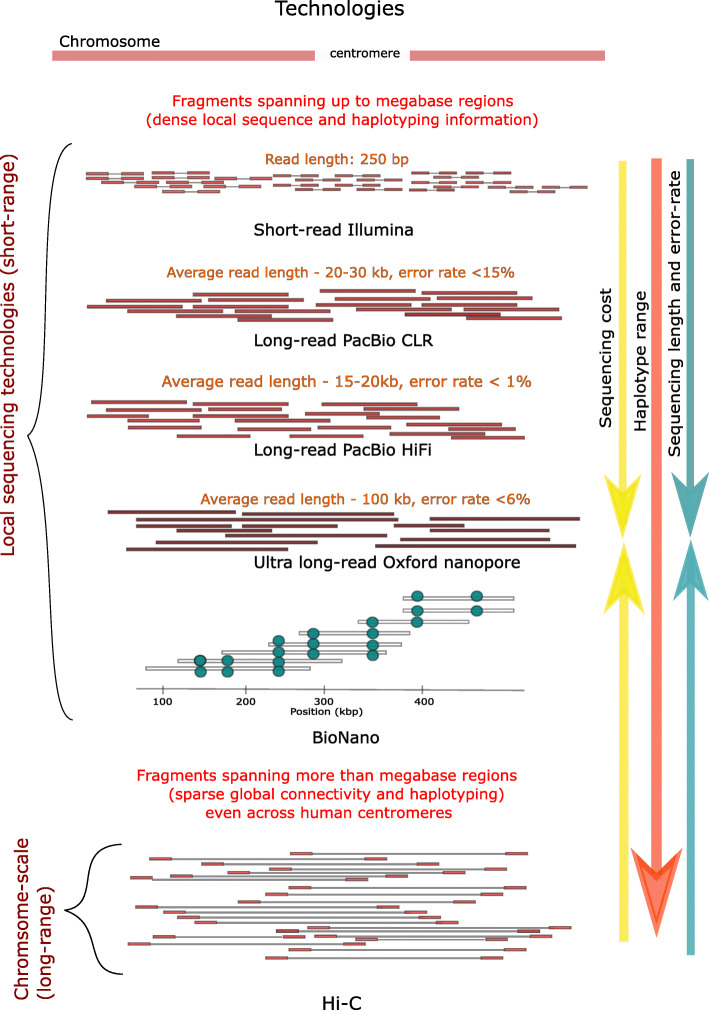
Third-generation sequencing technologies and their characteristics (read length, error rate and scale of information). The read length and scale of information (local versus chromosome-scale) together determine the haplotype range that can be achieved; moving down the schematic this range increases (orange arrow). Sequencing costs per sample increase moving from short-read sequencing down to nanopore sequencing, and then decrease again for BioNano and Hi-C (yellow arrows). Similarly, read length and error rate first increase moving down to nanopore sequencing, and then decrease again for BioNano and Hi-C (green arrows)

In the era of third-generation sequencing technologies, we define short-range (or local) sequencing that produces genomic fragments (reads) spanning up to megabases of the genome, but cannot connect across multi-megabase sized regions on the whole-genome scale. For example, long-read sequencing technologies such as single-molecule real time sequencing from Pacific Biosciences (PacBio) [30] and nanopore sequencing [31] (including ultra-long [32]) from Oxford Nanopore Technologies produce reads of the order of hundreds of kilobases in length, with error rates of 6–10% [33] (Fig. 1). The latest developments of PacBio’s HiFi technology can produce reads with an average read length of 15–20 kb at error rates similar to short-read sequencing (that is, an accuracy of > 99%) [34]. These advancements have made it possible to achieve near-complete human haplotypes that include microsatellites, repetitive elements, and other complex structural variations [35], which were previously inaccessible. In addition to these “true” long-read platforms, the Chromium technology from 10x Genomics [34, 36] (10xG) employs genome partitioning and barcoding to generate linked reads that span tens to thousands of bases. Finally, new optical mapping instruments from BioNano Genomics [37–39] can rapidly fingerprint megabase segments of a genome, enabling the detection of structural variation at a fairly low cost (Fig. 1). However, local (short-range) sequencing technologies suffer from inability to uniquely resolve near-perfect repeats above the size of their read length to produce full haplotypes. These limitations have necessitated the development of methods that can resolve haplotypes at the chromosome (or genome-wide) scale.

### Long-range sequence and haplotype information

Long-range (or chromosome-scale) sequencing consists of technologies that produce genomic fragments spanning across centromeres, thereby providing information to connect p- and q- arms over the entire genome, and connecting multi-megabases regions. Chromosome conformation capture methods such as Hi-C and related chromatin crosslinking protocols and produce long-range, mate-pair data for short-read sequencing [37] (Fig. 1). Hi-C technology [40] generates chimeric DNA fragments from two interacting chromosomal regions that are covalently linked together. These fragments are sequenced to produce paired-end reads representing genomic segments of a few kilobases and tens of megabases in physical distance.

Strand-seq is a recent single-cell sequencing advancement that allows independent sequencing of parental template strands and thereby characterization of individual homologues [41, 42]. Specifically, in the presence of bromodeoxyuridine (BrdU) during the DNA replication, sister chromatids generate one original template strand and one newly synthesized, BrdU-incorporated strand. The template strand and its directionality are preserved during the cell division phase that helps to separate the individual homologs.

These sequencing methods provide long-range information on genomic structure across centromeres, and they can be computationally assembled into chromosome-scale [43] at low cost. However, these haplotypes contain many gaps, especially in larger repeat regions. This limitation has led to further advancements in computational approaches for haplotyping (Table 2), such as the use of a hybrid approach that combines data from long-read and chromosome-scale sequencing technologies.

**Table 2:** Methods and computational tools for haplotype reconstruction

Approach	Tools	Data	Advantages	Disadvantages
*Reference-based phasing*				
Molecular haplotyping	WhatsHap [44], HapCut2 [45] and ProbHap [46]	Long reads such as PacBio, Hi-C of individual	Can phase de novo and rare variants	Limitations in complex regions such as centromeres, HLA, etc.
Single-cell phasing	CHISEL [47], Satas et al. [48], RCK [49]	Single-cell short-read	High precision at single-cell, detection of rare alleles	Engineering tricks required to scale to > million cells
Polyploid phasing	HapTree [50], Hap10 [51], WhatsHap-polyphase [52], H-PoP [53]	Local phasing	Can phase de novo and rare variants	Limitations in repetitive regions and not optimized for ploidy > 5
De novo *assembly*				
Diploid assembly	Falcon Unzip [23], Falcon phase [54]	Long reads and Hi-C of individual	Local phased contigs	No chromosome-scale assembly and computationally expensive
	DipAsm [55], Porubsky et al. [56]	Long reads and Hi-C of individual	Chromosome-scale diploid assembly	Collapsed assembly not suitable for repetitive regions
	Hifiasm, HiCanu [57], SDip [58]	HiFi reads of individual	High consensus accuracy and continuity	No chromosome-scale assembly
	pstools	Hifi and Hi-C reads	High-quality chromosome-scale haplotype assembly	Only designed for haplotyping diploids
	TrioCanu [59], Hifiasm+trio, WHdenovo [60]	Long reads of trios	Local phased contigs	Require family information
Polyploid assembly	SDA [61], SDip [58]	Long reads of individual	Local phased contigs	Need to be optimized for whole genomes
	POLYTE [62]	Illumina short reads	Local phased contigs	Does not scale well to whole genomes
*Strain-resolved metagenome assembly*				
De novo (re-) assembly	IDBA-UD [63], DESMAN [64]	Metagenome short reads	No prior knowledge required	Low sensitivity: rare haplotypes can remain undetected
	OPERA-MS [65]	Metagenome using short and long reads	High continuity	Computationally expensive
SNV-based assembly	ConStrains [66], StrainFinder [67], Gretel [68]	Metagenome short reads	Computational efficiency	Assembly accuracy depends on variant calling
Read binning	MetaMaps [69]	Metagenome long reads	Computational efficiency	Accuracy depends on database
Contig binning	ProxiMeta [70], bin3C [71]	Metagenome short reads and Hi-C	Reference-free, ability to link plasmids to host chromosome	Multiple technologies necessary (Hi-C + shotgun sequencing)

## Reference-based haplotype reconstruction

When a reference genome is available, haplotype reconstruction of the target sample comprises identifying co-occurring alleles of paternal and maternal copies over variant sites from sequencing data aligned to the reference. The process of obtaining these haplotypes is known as haplotype phasing [2].

Traditionally, reference panels of more than 100,000 individuals (large-scale projects such as UK10K) are genotyped and used to assign, probabilistically, the most likely local phase of the target sample based on the underlying evolutionary model [72–74]. This statistical phasing technique limits chromosome-scale haplotype [25, 75] production because the ancestry tracts from populations or the Mendelian laws of inheritance from trios have only local information to produce the haplotypes [72–74]. Phasing directly from sequencing reads, that is, the direct observation of two or more variants on a single molecule or in paired reads derived from the same molecule, overcomes the above limitations [50, 76]. The process of obtaining haplotypes directly from long-read and chromosome-scale sequencing data of a single individual—as opposed to phasing from genotypes by population inference or genetic analysis of pedigrees—is known as molecular haplotyping [2, 37, 77, 78]. Molecular haplotyping can produce chromosome-level phasing [79] that is highly accurate as determined by evaluation metrics (switch error rates and Hamming error rates < 1%). In molecular haplotyping, the key challenge is to disambiguate sequencing errors from true genetic variation.

### Diploid phasing

**Fig. 2:**
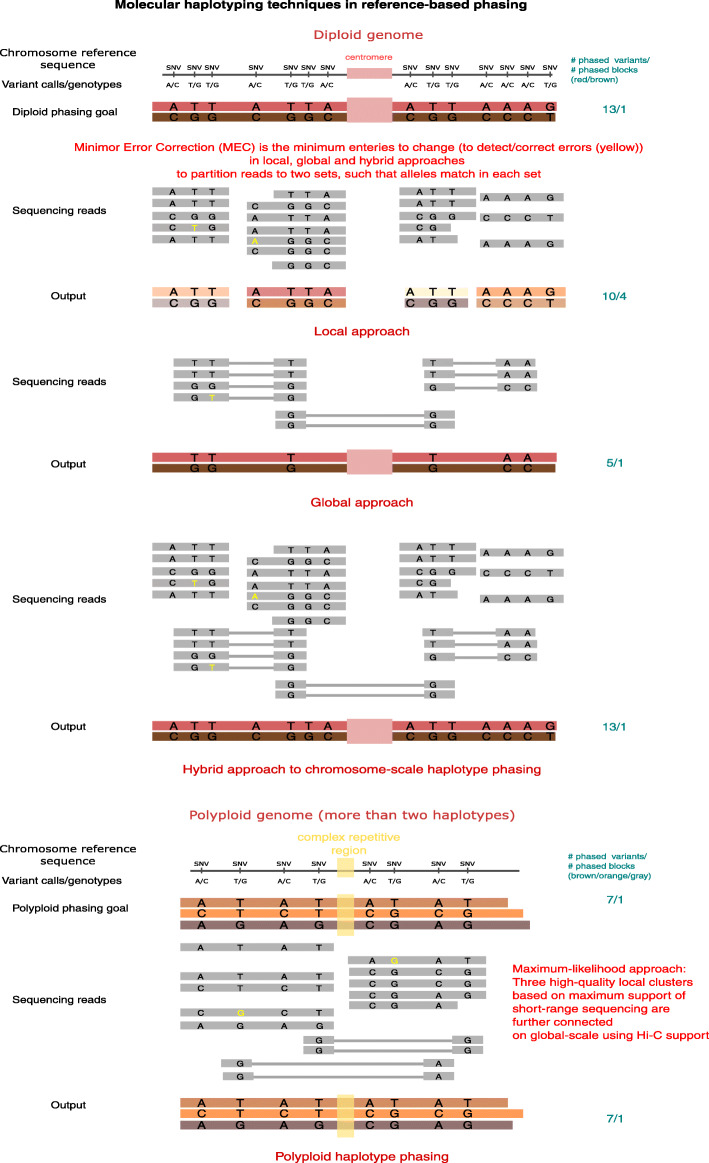
Molecular haplotyping techniques in reference-based phasing. Individual haplotypes are derived directly from sequencing data of the target sample based on read alignments to the reference genome. Local and chromosome-scale haplotype phasing make use of short- and long-range sequencing data, respectively; hybrid haplotype phasing combines the two data types

Reconstruction of haplotypes depends on how the heterozygous sites are connected on the chromosome-scale. If there are no reads that connect these sites, then the phasing is fragmented. Thus, the sites must be connected directly or indirectly via sequencing reads to achieve chromosome-scale phasing (Fig. 2). Long- and linked-read sequencing datasets, which span longer segments of heterozygous variants than short reads (Fig. 2), have improved the production of high-quality local phasing segments and the discovery of de novo and rare genomic variants.

The most widely used, state-of-the-art phasing methods are WhatsHap [44], HapCut2 [45], and ProbHap [46], which generate considerably longer haplotype blocks than short reads, in the order of several megabases in length with a switch error rate of < 0.5%. The performance of these methods is comparable and are optimized for different input data types, for example, all three methods produce comparable phasing completeness/accuracy/continuity using short-range sequencing, while HapCut2 and WhatsHap produce one large haplotype block on the chromosome-level using a combination of short-range and long-range sequencing data. The core aim is to assign all reads to two haplotypes while minimizing the number of sequencing error corrections or flips, also known as the minimum error correction (MEC) problem and weighted minimum letter flip (WMLF) problem [80]. More specifically, the MEC formalism, which is the most widely used, is the process of finding the minimum cost of correcting the sequencing data to partition the read set into two homologous sets such that the alleles between any two reads in any partition match [22, 44]. The MEC formulation is NP-hard [80, 81]. In practice, this formulation is solved using computational techniques such as dynamic programming, probabilistic modeling, graph-based optimization, and linear programming [82]. To scale these algorithms to human-sized genomes and beyond, a combination of greedy heuristics and dynamic programming is prominent [45, 83].

Genome-wide molecular phasing, a task that computes combinatorial solutions at chromosome-scale by using long-range sequencing technologies such as Hi-C and Strand-seq (Fig. 2), is a more challenging algorithmic task. Computational tools used in practice, such as HapCut2 [45] and StrandPhaseR [79], reduce the search space using greedy heuristics based on the MEC formulation. Remarkably, these tools generate haplotype blocks spanning full chromosomes [45]. However, they can typically phase only 50–70% of variants using Hi-C/Strand-seq [48]. This phasing sparseness can be improved with uniform-coverage data that is often difficult to produce experimentally. In this new era of advancements across technologies, hybrid algorithms that combine different data types at local and genome-wide scale are prominent. For example, WhatsHap [44] and HapCut2 [45, 84] both have local as well as chromosome-scale phasing modes. In addition, WhatsHap [44] can perform family-based phasing, which has been shown to give better results than single-individual approaches in terms of accuracy and phasing completeness [85]. The disadvantage is the unavailability of trio sequencing data for various species.

Hybrid approaches (combining long or 10xG reads with Strand-seq or Hi-C datasets [79]) for single individuals are leading the way into production-level efforts and provide competitive phasing performance at chromosome-scale with hamming error rates < 1% and switch error rates < 0.5% by using ~30x HiFi or 10x linked-reads combined with ~30x Hi-C (Fig. 2). State-of-the-art hybrid phasing tools are WhatsHap and HapCut2. HapCut2 directly works on reads from long-read and Hi-C sequencing using likelihood inference optimization, while WhatsHap operates on Strand-seq haplotypes and long-read reads using MEC formulation. These methods have enabled impressive advances in the production of high-quality chromosome-scale phasing, for example, phasing Ashkenazi, PGP-1, Chinese human genomes [15, 86], as well as genomes from the 1000 Genome project for a comprehensive SV callset.

Beyond the above bulk sequencing methods, single-cell phasing [76] has recently been used to study single-cell genomic heterogeneity. However, extremely low sequencing coverage (< 0.05x per cell) has restricted its use in phasing of large multi-megabase segments in individual cells for genome-scale analysis. Recent single-cell phasing methods such as CHISEL [47], Satas et al. [48], and RCK [49] use probabilistic models at a single-cell level that have the advantage of haplotyping rare alleles, which can be used to determine local relationships in allele-specific somatic aberrations, but cannot phase all variants across the genome. Thus, in the near future, combining single-cell and bulk sequencing approaches for phasing may enable accurate and complete genome-wide characterization of genomic heterogeneity, including rare alleles and cancer genomes.

### Polyploid phasing

In phasing diploid genomes, the haplotypes are complementary: given the genotype data, determining one haplotype sequence directly identifies the other. However, polyploidy is common in plant genomes, and in the case of a *k*-ploid sample, *k*–1 haplotypes need to be computed before the final haplotype can be inferred. For example, there are k! possibilities (instead of two in diploid) to connect a pair of SNPs in the polyploid. A higher number of haplotypes also requires a greater overall sequencing depth, resulting in a larger number of reads per genome to be processed. This additional complexity requires specialized, highly optimized algorithms to resolve polyploid phasing (Fig. 2).

To solve polyploid phasing problems, the maximum likelihood framework is a common algorithmic strategy. HapTree [50, 87] uses the relative likelihood algorithm to identify k-ploidy phasing for first *n* SNPs, that is conditioned on previous *n*-1 SNPs. This approach lays the first theoretical foundation of polyploidy phasing problems. A few works have attempted to formulate the problem using approximate MEC formulations, for example, SDhaP [88] solves approximate MEC using semidefinite programming, and H-PoP [53] partitions the reads into haplotypes by solving a generalization of the MEC problem. However, there is an inherent problem in MEC based methods that it leads to inaccurate phasing as demonstrated by Motazedi et al. [89].

To address these shortcomings, local phasing methods such as Ranbow [90] follows graph-based algorithms by leveraging allele co-occurrence in overlapping short reads to produce accurate polyploid phasing, but lacks in haplotype block N50 length. An alternative phasing approach, designed specifically for long-read sequencing, is WhatsHap-polyphase [52] (available as part of the diploid phasing tool WhatsHap) that produces accurate phasing (switch error rates < 1% and hamming error rates < 2%) and better N50 compared to short-read methods. A recently linked-read based method [51]: Hap++ and Hap10 produces slightly more accurate and comparable haplotype block N50 compared to WhatsHap-polyphase, at the cost of efficiency. However, these methods have limitations to produce chromosome-scale haplotypes.

Similar to diploid phasing, additional long-range information can allow for chromosome-scale haplotype reconstruction of polyploid genomes. For example, TriPoly [91] uses family information (parent-offspring trios) to infer haplotypes from either short- or long-read sequencing data. This results in larger haplotype blocks compared to other approaches, in particular in regions with low divergence between haplotypes.

While these methods represent an important step forward in polyploid phasing, hybrid methods that leverage HiFi, or alternatively linked-read data, and long-range Hi-C data can potentially produce chromosome-scale haplotypes for complex repetitive polyploids. In the near future, further algorithmic developments in local k-mer strategies and graphs-based approaches that focus on every haplotype could enable chromosome-scale phasing in large polyploid genomes.

## De novo haplotype assembly

De novo genome assembly exploits the overlaps between sequencing reads, without any bias towards reference sequence. The main steps in a standard genome assembly workflow are sequence graph construction, error correction, contig formation, scaffolding, and polishing of the assembled sequences [92]. The most widely used assembly software is Canu [93], FALCON [94], Flye [95], wtdbg2 [96], and shasta [97]; we refer the reader to a review by Sedlazeck et al. [98] for a literature survey on genome assembly using the latest technologies. Of particular interest to haplotype assembly are assemblers specifically designed for PacBio HiFi datasets, which, for the first time, improved the per-base quality of assemblies dramatically [99] and reduced the need for computational intensity of the error correction step.

After contig construction, the next step is to create scaffolds by ordering and orienting contigs along the chromosomes using chromosome-scale information sources, such as Hi-C data. Scaffolding with the chromosome-scale data types has resulted in chromosome-scale consensus assemblies of human genomes.

Due to sequencing errors, the reads often undergo error correction before contigs are formed; this is particularly relevant when using error-prone long-read sequencing technologies. Despite the error correction process, contigs and scaffolds may still be erroneous and thus another round of error correction is performed (now referred to as polishing) using tools such as Racon [100].

However, for diploid and polyploid genomes, most standard de novo assemblers collapse the haplotypes into a single consensus sequence, but it is important to realize that all haplotype information is ignored. Nevertheless, the consensus assembly is useful for de novo haplotype assembly approaches as discussed below.

**Fig. 3:**
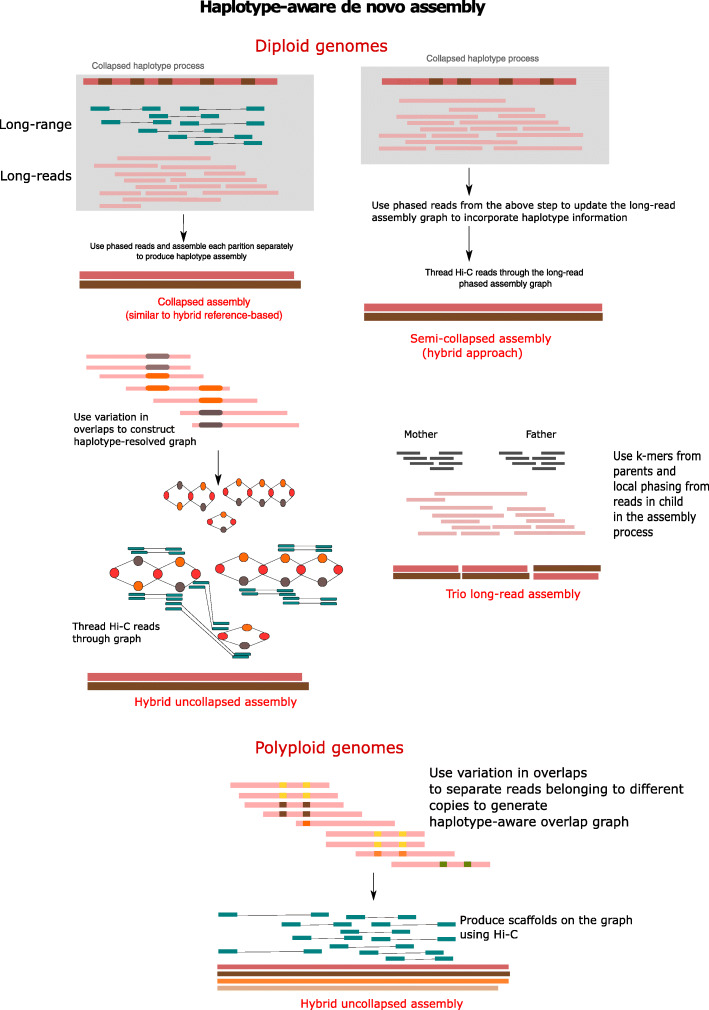
Haplotype-aware de novo assembly. Collapsed assembly approaches identify sequence variants on a consensus assembly and subsequently phase these variants into haplotypes using chromosome-scale data (Hi-C). Semi-collapsed approaches follow a similar approach, but after phasing, variants in the initial assembly graph are updated and final contigs are produced based on this updated graph. Uncollapsed approaches directly determine haplotype-specific overlaps in local sequencing reads by retaining SNPs and repeat variation in all possible overlaps and construct haplotypes based on the selected overlaps

Reconstructing every individual haplotype from sequencing data instead is known as de novo haplotype assembly (Fig. 3) and is even more challenging than consensus generation (de novo assembly) due to varying repetitive and heterozygosity rates, noisy sequencing data, chimeric reads, insufficient read length, and non-uniform coverage.

In de novo haplotype assembly, there are two major related challenges: finding ordering of sequencing reads and distinguishing reads to haplotypes. To find ordering of reads, the brute-force approach is to align all reads to all other reads, where the performance is directly proportional to the square of the number of reads. In repetitive regions, finding alignments of reads is even more expensive. For systematic study, overlap-based [101] or de Bruijn graph [102]-based techniques are used. To solve another challenge of finding haplotype of reads, the commonly used approach was heterozygous SNPs informative sites to partition reads to haplotypes in the space of single consensus sequence (due to high error rates in long-read PacBio and ONT data); however, latest advancements in Hifi allowed to separate reads to haplotypes during the overlapping step as discussed below.

### Diploid haplotype assembly

Algorithms for long-read sequencing are now able to produce megabase contigs for haplotypes and improve the availability of reference-quality genomes for humans and various other eukaryotic organisms [23, 103, 104]. This technique has been applied to assemble phased sequences of humans [55, 103] (Table 1), diploid potato [105], zebra finch [54], cattle [54], and goat genomes [106]. Broadly, the bioinformatic approaches for diploid assembly fall into three classes: collapsed, semi-collapsed, and uncollapsed (Fig. 3).

In collapsed diploid assembly, generic de novo assemblers are used to generate a consensus sequence. Subsequently, by using heterozygous SNP information from reads aligned to the consensus sequence, long-read and chromosome-scale sequencing reads are partitioned into haplotype-specific read sets, which are then separately assembled into haplotypes. This technique is used by tools such as DipAsm [55] or Porubsky et al. [56], resulting in phased contigs of up to several tens of megabases and chromosome-scale phased scaffolds, with haplotype sizes of ~ 3 Gb each and overall base quality scores of >Q48. The preferred input data types are PacBio Hifi and Hi-C. This technique works well for the human genome in regions of low heterozygosity, but fails in highly repetitive and high heterozygosity regions.

Alternatively, the widely used FALCON-unzip [23] method uses a semi-collapsed approach for diploid assembly from long noisy reads, where the initial assembly graph is generated using FALCON and a consensus sequence is generated. Similar to the collapsed approach, reads are partitioned into haplotype-specific sets using SNP information. Phased read information is then used to update the initial assembly graph, and phased contigs (size of about several tens of megabases with quality score < Q48) are reported [94]. These phased contigs are then combined into scaffolds using phase information (> 1 Mb) provided by ultra-long nanopore or Hi-C data, as employed by FALCON-Phase [54], producing a chromosome-scale diploid assembly. The preferred input data types are PacBio CLR and Hi-C. Similar to the collapsed approach, it works particularly well for human genomes when the heterozygosity rate is low, but fails in regions or genomes with high repeat and heterozygosity rates. However, the most promising uncollapsed approaches overcome these limitations by directly determining haplotype-specific overlaps in the overlap step of graph generation using SNP information from overlapping reads [58]. The core idea is to preserve heterozygosity and repeat information from various data types in the graph space. To achieve this, on every reference read, similar reads from the same haplotype and repeat are detected based on shared alleles at SNP sites and are clustered together. Standard tools use run-length encoding or base-level alignment [57] in the overlap step. Thus, a haplotype and repeat-aware overlap graph is generated with subsequent graph cleaning steps, finally reporting phased contigs.

The recent invention of PacBio HiFi technology has made the diploid assembly process, that entains ordering as well as the phasing in the assembly process, easier [58]. A whole generation of new algorithms based on uncollapsed approaches have become possible due to the availability of accurate long-read data and are implemented in tools such as Hifiasm (https://github.com/chhylp123/Hifiasm), HiCanu [57], and SDip [58], producing contigs with lengths of several tens of Mb having base quality scores >Q50, but phased blocks of only a few hundreds of kb. In these systems, the field is moving towards accurate HiFi data using *k*-mer based strategies for haplotype-aware error correction of phased contigs, which can be completed in a few hours for human-scale genomes. Similar to semi-collapsed approaches, these phased contigs can be combined into phased scaffolds using long-range information to produce a chromosome-scale diploid assembly. For phased scaffolding, one of the largest challenges is the development of computational models that combine both phasing and scaffolding information together, an approach that is recently explored by the pstools method (https://github.com/shilpagarg/pstools) in the graph sequence space. The preferred input data types are PacBio Hifi and Hi-C.

When trios are available, methods such as TrioCanu [59] search for k-mers from maternal and paternal haplotypes in reads that were sequenced from the child to produce haplotype-specific read sets and then assemble these separately. New methods (Hifiasm+trio and WHdenovo [60]) use both trio and local phasing information from sequencing data, thus resulting in high-quality phased contigs. Although pedigree-based haplotype assembly allows for improved accuracy as compared to haplotype assembly of individuals, it requires sequencing of three individuals which limit its applications. Moving forward, substantial improvements to the uncollapsed approach using graphs and k-mers from local and chromosome-scale sequencing datasets in single individuals are expected to become routine for chromosome-scale diploid assembly within the next few years.

### Polyploid haplotype assembly

Polyploid assembly is in principle an immediate extension of diploid assembly; however, an increase in the number of haplotypes inflates the search space dramatically on the whole-genome scale. Some progress has been made in computational approaches for local haplotype assembly in polyploids as a potential step towards chromosome-scale polyploid assembly. For example, using Illumina short-read sequencing reads, POLYTE [62] performs overlap graph-based de novo assembly for diploids and polyploids. Since short reads cannot span difficult-to-assemble regions such as long repeats or variant deserts, the haplotype-specific contigs produced by this algorithm remain relatively short, yet highly accurate. Alternatively, linked-read technologies have been used to obtain long, contiguous, polyploid genome assemblies [107]. Long-read based methods such as SDA [61] and SDip [58] have demonstrated their ability to assemble polyploid regions in human genomes several megabases in length (Fig. 3). With some further algorithm engineering, it should be feasible to apply these methods to obtain chromosome-scale haplotypes of polyploid genomes with chromosome sizes of less than tens of megabases in size. However, for large complex repetitive polyploid genomes, algorithm development is required to exploit the latest HiFi sequencing, and its combination with other technologies such as Hi-C, by separating all haplotypes during the assembly process in graphs on the whole-genome scale.

## Strain-resolved metagenome assembly

**Fig. 4:**
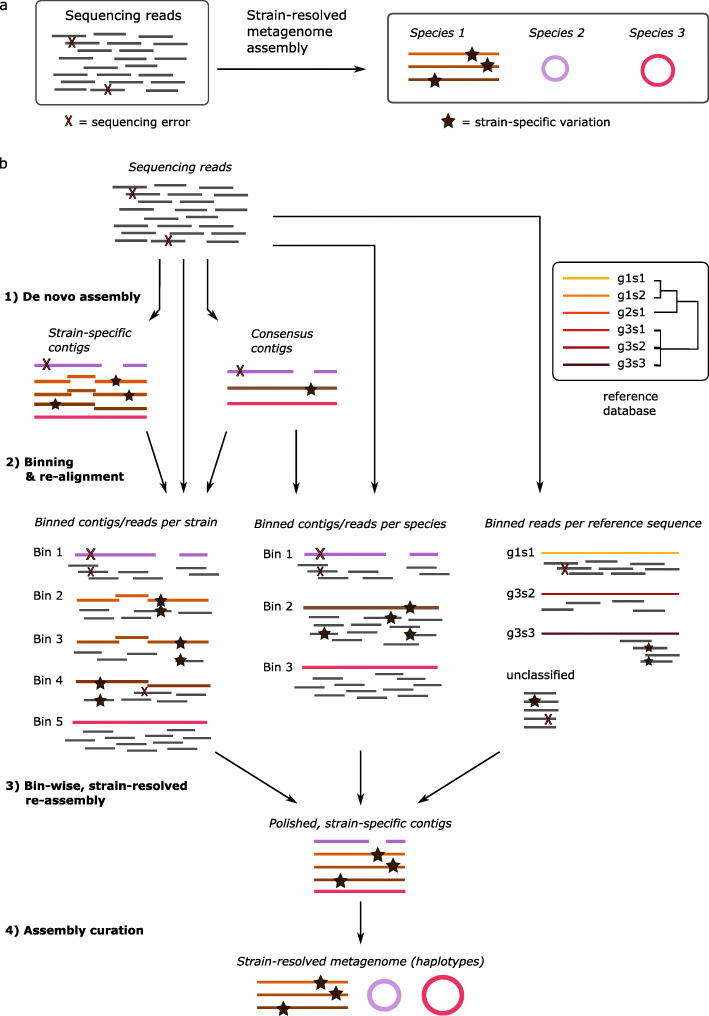
Strain-resolved metagenome assembly. **a** Given a pooled sequencing sample, the goal of strain-resolved metagenome assembly is to reconstruct all individual microbial strains. **b** A typical workflow consists of four steps: de novo assembly, contig binning, bin-wise re-assembly, and assembly curation. Each step can be performed at the species-level or at the strain-level, as illustrated in the left and middle column, respectively. Some workflows skip the initial de novo assembly step and perform strain-resolved binning directly on the sequencing reads, which can be reference-guided (right column)

Haplotype reconstruction plays an important role in strain-resolved metagenome assembly, that is, the computational reconstruction of haplotypes from pooled sequencing to identify microbial strains (Fig. 4a). Variation within and across species and low per-strain (haplotype) sequencing depth across different datasets make it extremely difficult to distinguish genetic variation from sequencing errors [108, 109]—a microbial sample can contain several hundreds of haplotypes with levels of variation ranging from < 1 to > 5%. Similar to de novo haplotype assembly for diploid and polyploid genomes, additional hurdles are the longer repeats and homologous regions between closely related strains (that is, intergenomic repeats) relative to sequencing read lengths.

The bioinformatic approaches for metagenome assembly are highly related to haplotype reconstruction in diploids and polyploids, as noted by Kolmogorov et al. [110] and Nicholls et al. [111]. In practice, various diploid or polyploid haplotyping approaches are adapted to solve the strain-resolved metagenome assembly problem [67, 68, 110] and vice versa [62]. Here, we classify two classes of methods: species-level and strain-resolved metagenome assembly—species-level reconstruction aims at constructing a single (consensus) haplotype per species, while strain-resolved assembly aims at every strain of species. Each class of methods can further be distinguished into reference-based (database of species/strains) and de novo approaches—similar to diploids and polyploids approaches. The advantage of reference-based approaches is that they are efficient, but they often lead to biases towards the database(s) being used, for example, due to incompleteness of reference databases, as many microbes on Earth remain uncharacterized [112]. This type of reference bias is even more pronounced than the reference bias observed in diploid and polyploid assembly, hence de novo algorithms are an essential component of any complete, unbiased analysis of metagenomes.

From an author’s perspective, we present a general workflow for metagenome assembly that consists of several steps [113–116] (Fig. 4b): (1) de novo metagenome assembly to produce contigs or scaffolds; (2) contig binning per genome, either de novo or reference-guided; (3) mapping reads back to individual bins and reassembling each bin; and (4) curation of the resulting assembly per bin. In theory, each of these steps can be performed at the species-level or at the strain-level, depending on the goals of the study.

### Short-read metagenome assembly

The commonly used data structures for metagenome assembly are de Bruijn and overlap graphs with special tuning of parameters related to sequencing depth, variations, and errors. For example, IDBA-UD [63] is the first metagenome assembly method based on de Bruijn graphs, and LSA [117], a method that uses *k*-mers to identify (partial) bacterial strains in short-read sequencing data with relative abundances as low as 0.00001%. Other de novo approach tools are MEGAHIT [118] and metaSPAdes [119], but these can only produce species-level assemblies. Alternatively, several approaches are based on single-nucleotide variants (SNVs), which are identified using metagenome assemblies or reference databases, or entirely de novo—see REF [120] for a detailed review. The major limitation of such approaches is that structural variants are completely ignored. Available methods for SNV-based metagenome assembly with strain-resolution include ConStrains [66] and StrainFinder [67], both of which can trace strain identities across multiple samples (a longitudinal time series). Recently, a Bayesian model for local haplotype reconstruction was proposed in a promising approach called Gretel [68], which is based on a new data structure designed to efficiently store variation across sequencing reads. All of these methods (ConStrains, PathFinder, Gretel) aim at strain-level sensitivity in step 1 (Fig. 4b). Another class of methods achieve strain-level sensitivity in step 3 while relying on species-level sensitivity described in steps 1 and 2 (Fig. 4b). DESMAN [64] is one such method, which leverages base haplotype frequencies in a Bayesian model. Finally, if strain-level assembly is not achieved in steps 1–3, further curation in step 4 can help to identify intra-species variation [121] (Fig. 4b).

These short-read methods take an important step in strain-level metagenome assembly field and are widely used in studying the human microbiome, health and disease [122, 123], as well as the biodiversity of marine ecosystems [64]. These methods establish the first step towards producing chromosome-scale metagenome assembly.

### Hybrid metagenome assembly

Local and chromosome-scale sequencing is essential in achieving chromosome-scale, strain-resolved metagenome assemblies. Recently, a hybrid metagenomic assembly approach (OPERA-MS) was proposed that combines short-read contig assembly with long-read scaffolding and binning to obtain high-quality, strain-resolved metagenomes [65]. OPERA-MS provides an order-of-magnitude improvement in contiguity compared to short-read metagenomic assemblers and a 200% increase compared to generic long-read assemblers. As little as 7x haplotype coverage with long reads was sufficient to obtain megabase N50 genomes [65]. Alternatively, the first long-read metagenome assembler (MetaFlye [110]) proposes the use of local k-mer distributions to identify species of low abundance. MetaFlye can assemble haplotypes with as little as 10x per-haplotype coverage [110], though the extent to which it can distinguish between closely related strains remains to be evaluated. Another approach by Anoton et al. [124] uses long-read assembly (with MetaFlye), followed by assembly curation using short- and long-read data. Yet, another approach, MetaMaps [69], offers strain-level long-read binning, but this requires a reference database and therefore complicates discovery of new haplotypes (Fig. 4b).

Alternatively, the combination of Hi-C and shotgun sequencing enables chromosome-scale, strain-resolved metagenome assembly through improved clustering of metagenome-assembled contigs at strain level, as well as linking of plasmid sequences to the chromosomes of their hosts [40, 70, 71]. Such an approach has recently been used to leverage structural information obtained from Hi-C data of the human gut microbiome to perform strain-level assembly and enable tracking of microbial evolution over time [125].

For complex repetitive metagenomes, HiFi reads, in combination with Hi-C, have the ability to become the strategy of choice to produce complete, strain-level resolved metagenome assemblies in the near future.

## Remaining challenges and perspectives

### Repetitive regions

Haplotype reconstruction remains challenging in multi-megabase complex repetitive regions. Despite considerable time and effort, the current version of the human reference genome either contains gaps or is collapsed in these regions without haplotype-level resolution. These regions include tandem repeats [126], segmental duplications [127, 128], sex chromosomes (containing complex heterochromatin repeat structures) [129, 130], the mitochondrial genome [131], pseudo-autosomal regions [132] (PARs), centromeres (or pericentromeric regions) [133], ribosomal DNAs [134] (or acrocentric regions), and subtelomeric regions [135]. For example, the human genome includes complex satellite arrays of repeats in centromeres. α-satellite DNA contains ∼171-bp tandem repeats that are organized into higher-order repeats (HORs), with a single repeat structure reiterated over hundreds or thousands of times with high (>99%) sequence conservation [136]. Some human chromosomes comprise ~3200 repeats of ~2 kb HORs and ~1,100 repeats of a 1.8-kb HOR unit [137]. The centromere assembly produced by state-of-the-art tools (centroFlye [138], HiCanu [57]) using HiFi and ultra-long nanopore reads is haploid [20]. Humans are diploid and should produce two haplotype sequences in centromeres; however, currently, there are no algorithms, technologies, or tools to achieve this goal.

Recent developments in long, accurate long reads (Hifi) as well as ultra-long nanopore reads could pave the way for new advancements in finishing centromeric and other highly repetitive regions in humans and polyploids. Computationally, Hifi reads can be decomposed into monomers [139] that are represented in the graph, where monomers are nodes and edges represent the adjacencies of node sequences from reads. In this process, the haplotype variation is also considered in the monomers that can result in a haplotype-aware graph. Through this graph, the ultra-long nanopore reads are anchored to potentially find ordering between repeating units and disentangle the graph. On a complex centromeric region involving >2000 repeat units, the in situ information such as chromosome visualization [140] can further be helpful to order the repeating units. Further increase in read lengths to several megabases in size, and/or reads with spatial coordinates, and/or longer reads with > 99% accuracy, as well as innovations in k-mer and graph-based strategies to distinguish variations/motifs may enable exploration of high-resolution haplotypes in these human centromeric regions.

### Scale

Developments are required to scale haplotype reconstruction efforts to overcome current limitations and enable routine application to more than hundreds of genomes at a time. Such developments require innovations in technologies that are cheaper and easy to use than long-read, HiFi and Hi-C sequencing. Alternatively, a further reduction in sequencing costs of existing technologies will be required to scale up efforts.

With an exponential growth in datasets, the real challenge will be to store and access haplotyping data in an efficient way, which can potentially be achieved by applying massive parallelism (detailed reviews in [141, 142]). In addition, cloud-based strategies will be required for storing, accessing, and sharing data (for example, https://vgp.github.io/genomeark/). Building a collaborative haplotyping platform that can serve as a repository of data and computational tools and enable exchange of ideas for the scientific community may help to usher in a new era of biological discoveries.

Further integration of datasets using scalable bioinformatics approaches will be important. Innovative algorithm engineering (for example, using sequence sketches instead of full sequences has been shown to vastly reduce storage and memory requirements [143]) could enable production-level integration of datasets for haplotype reconstruction. Beyond engineering efforts, combining reference-based and de novo approaches will improve scalability. More specifically, genomes that are similar to known samples can be reconstructed efficiently using reference-based approaches, thus reducing de novo efforts to the remaining highly divergent genomes.

### Validation, benchmarking, and annotation

For the final haplotype assemblies of diploid genomes, many high-quality benchmarks are available, and validation is done with standardized evaluation metrics as a standard practice for non-repetitive regions. This is not the case for polyploids, tumors, and complex repetitive regions in diploids. Innovations in algorithms (beyond *k*-mer approaches) that improve the capability for assessment and biological validation could benefit from a public collection of high-quality benchmarks, for example in the form of a community-driven assessment initiative similar to the Critical Assessment of Metagenome Interpretation [108] (CAMI), Assemblathon [144, 145], and Genome Assembly Gold-standard Evaluations [146] (GAGE). As the field advances to produce high-quality chromosome-scale phased sequences, the next critical step will be in the development of new gene annotation tools [147] to enable more precise downstream analyses in the coming decade.

### Visualization

Another challenge is the visualization of large-scale haplotyping raw sequencing datasets and haplotype sequences from multiple species. The combination of long haplotype sequences and divergence across or within genomes and the large diversity of haplotyping data types pose numerous visualization challenges. While a number of tools exist (reviewed in [148, 149]), none can be used to visualize large-scale phased sequences. New visualization techniques will be required that enable abstractions or reductions in data dimensions from multi-scale, multiple data measurements, binary encoding of variations and divergence across haplotypes for visual maps, and discovery of informative patterns in the haplotyping data. Interactive visualization or animation in the chromosome-scale coordinate system can be useful.

## Conclusions

Chromosome-scale haplotype reconstruction has yielded new insights into the genetic underpinnings of disease pathogenesis, evolution, and comparative biology. To overcome the limitations of sequencing reads to cover genomic repeats, chromosome-scale haplotype reconstruction using a combination of long-read (HiFi and ultra-long ONT) and chromosome-scale sequencing (Hi-C) datasets, along with integrative algorithms, has become a common strategy to produce haplotypes in diploids, but not polyploids yet.

Improvements in fragment lengths and combining complementary technologies through innovative algorithms (graphs, *k*-mers and data-driven) will be state-of-the-art to reconstruct high-quality haplotypes with fewer gaps in the near future. Both fragment accuracy and length—a few megabases size with accuracy of > 98%—could be important to finish haplotypes. Major reductions in sequencing and computing costs will be critical to scale efforts to thousands of genomes at a time. In the next decade, algorithmic and technological advances, paired with the incorporation of haplotypes with disparate layers of biological information, could mark a new era of gapless end-to-end haplotypes and further our understanding of complex biological phenomena.

## Supplementary Information

**Additional file 1.** Review history.

## Glossary

Barcoding: Labeling reads with barcode sequences to identify fragments from the same partition
Base-level alignment: Position-wise alignment of nucleotides in a pair of sequences
Chromosome-scale haplotype: Nucleotide sequence spanning a full chromosome for a given homologous copy across centromeres
Compound heterozygosity: The phenomenon where a combination of recessive alleles for a given locus harboring different mutations together can cause genetic disease
Pan-genome graph: A data structure that contains sequences shared across multiple genomes of species. The differences in genome sequences are also stored
Diploid: A genome containing two complete sets of chromosomes, one from each parent
Aneuploidy: Normal human cells contain two chromosomes. Aneuploidy is the phenomenon of increase or decrease in chromosomes in cancer cells compared to normal cells
Dynamic programming: A mathematical optimization approach where the problem is recursively divided into subproblems whose solutions build towards a global, optimal solution
Optical mapping: A technique for constructing ordered restriction maps of the whole genome, called optical maps, by locating restriction enzyme sites on an unknown genomic sequence
Genome partitioning: Using microfluidics to physically separate genomic sequences
Greedy heuristic: Solving an optimization problem by finding a locally optimal solution
Heterozygosity rate: Rate of mutations (differences) between haplotypes
k-mer distribution: Frequency distribution of substrings of length *k* from an original sequence
Long-range promoter-enhancer interaction: Transcriptional enhancers interacting with their target-gene promoters over a considerable genomic distance, affecting gene expression
Mate-pair sequencing: Generating paired-end (short) reads with particularly long inserts to span a large genomic region
Haplotype block: A genomic region on the chromosome in which a series of SNPs (or genetic loci) are phased together
Scaffolds: Scaffolds are the sequences produced by ordering the contiguous sequences with their correct orientation
Variant deserts: Genomic regions with a fewer variants compared to an average
NP-hard: The complexity class of decision problems that are intrinsically harder than those that can be solved by a nondeterministic Turing machine in polynomial time
Sequence sketches: Sketching of genomic sequences is the process of indexing and hashing the data for faster direct access and efficient memory usage
Hamming error: It is an evaluation metric to compare the binary strings. This metric is used to evaluate the long-range phasing on the chromosome-scale level
Switch error rate: Number of switches between true and alternative haplotypes, relative to the number of variant positions
Base quality: Number of erroneous bases relative to total assembly length (can evaluate mismatch errors and indel errors separately or jointly)
Phased contig: Contiguous nucleotide sequences that represent a subsequence of a haplotype
Phased scaffold: Haplotype sequence linking phased contiguous sequences (contigs) originating from the same haplotype, separated by gaps of known length
Polyploid: A genome containing more than two sets of chromosomes; this is common in plant species
Metagenome: The collection of genomes of many species as well as their strains present in an environmental sample.
Run-length encoding: A form of lossless data compression in which each repetitive sequence is stored as a single repetitive element along with its number of consecutive occurrences, rather than the whole repetitive sequence
Variant calling: Variant calling is the process of finding genomic variation (mutations) from sequencing data aligned to the reference genome
