# Supplementary material for: Computational methods for chromosome-scale haplotype reconstruction
Source: Genome Biol. 2021 Apr 12;22:101. doi: 10.1186/s13059-021-02328-9 (PMC8040228; doi:10.1186/s13059-021-02328-9)
Supplement: Supplementary file 1 — Additional file 1. Review history. [file 13059_2021_2328_MOESM1_ESM.docx]

Review History

**First round of review**

**Reviewer 1**

**Comments to author:**

In this review, Shilpa Garg summarized recent progress towards whole-chromosome scale haplotype reconstruction and assembly, focusing on computational approaches, and then discussed applications and limitations of these methods. Although there has been some excellent reviews on haplotype-resolved genomic analysis, e.g., Snyder, Adey et al., Nature Review Genetics 2015, the topic of whole-chromosome haplotype analysis that is the focus of the current review should benefit the general audience.

While I enjoy reading the review, I feel the following areas can be improved with some additional discussion/clarification.

Comments:

1. The motivation for deriving whole-chromosome haplotype knowledge and its immediate applications weren't clear based on the Introduction. The various applications listed by the author, including complex genomic variation, variants in regulatory elements and allele-specific gene expression, and evolutionary biology, have been largely restricted to locus-specific analysis but not reaching the whole-chromosome scale. It is unclear how whole-chromosome haplotype analysis may transform these areas of research or what new questions will be enabled by whole-chromosome haplotype knowledge. On a minor point, Ref.11 was not related to cancer genome analysis even though the approaches developed in this study may be applicable. The immediate applications of whole-chromosome haplotype knowledge in cancer genome analysis is also not obvious given that the prevalence of allelic imbalance in aneuploid genomes can enable whole-chromosome scale haplotype phasing without using any of the recent methods.

2. The author discussed both technological advances and computational innovations that are necessary to make whole-chromosome scale haplotype reconstruction feasible. For technological advances, the author mentioned "short-range sequence information" from "long-read" sequencing; this may confuse the general audience who will associate "short-range sequence information" with illumina short-read sequencing. It will be useful to define "short-range" sequence information and explain why "long-read" sequencing only gives "short-range" sequence information and but not "long-range" sequence information. All the technologies mentioned by the author, including PacBio SMRT, 10X genomics, and BioNano optical mapping, are commonly referred as "long-range" sequencing.

3. For long-range sequence information, the author mentioned both Hi-C and Strand-Seq. Whereas Hi-C can be implemented both on bulk samples and in single cells, Strand-Seq strictly requires cycling single cells. Both technologies provide long-range sequence information, but their utilities are differentiated by the experimental procedure. The author should discuss the sample requirements of these technologies and their implications for sequencing applications. It is also useful to discuss similar requirements for sequencing technologies that the author listed under "short-range" sequence information.

4. The section on Reference-based haplotype reconstruction includes both statistical phasing based on "reference haplotype data" from large cohorts, and molecular haplotyping that is based on long reads aligned to the reference genome. The section title causes confusion between these two applications. I also found the author's discussion of statistical phasing to be quite superficial. Perhaps the author can simply point to the original studies (Ref.33-36) and briefly describe why statistical phasing cannot reach whole-chromosome scale.

5. In the section titled "diploid phasing", the author listed several methods including WhatsHap, HapCut2, and ProbHap. These methods are applicable to different types of sequencing data and can generate haplotype inference with different completeness and accuracy depending on the input data. Given the focus on computational methods, the author should discuss these differences and how these methods are optimized for different input data.

6. I like the author's description of the comptuational algorithms of diploid phasing. However, the author's statement that "HapCut2 can typically phase only 50% of variants (Ref.42)" is misleading. I think the author was referring to HapCut2 can only phase 50% of variants using Hi-C data of 40x (Edge et al. claimed that HapCut2 can phase 60-70% of all variants). It is necessary to describe the input data for the quoted benchmark and comment on whether this can be improved with more data.

7. For Hybrid approaches, the author should describe what input data are required to achieve accurate whole-chromosome haplotype inference (hammming error < 1% and switch error rate < 0.5%), including the type of long-range sequencing data, depth requirement, variants etc. The examples of phased Ashkenazi and Chinese human genomes are not convincing as "impressive advances in the production of high-quality chromosome-scale phasing," as these genomes had been phased by trio sequencing and are often used for optimizing the computational methods. In addition, the author should cite the study by Bansal, Bioinformatics 2019, which combines reference-based phasing and Hi-C haplotyping to achieve whole-chromosome scale haplotype inference. The utility of this method appears to be broader than the others as it only uses one data type.

8. For single-cell phasing, the first paper that demonstrated its utility is Ref.37 but that was not cited appropriately.

9. The author gave a nice introduction on polyploid phasing but I am not sure whether whole-chromosome haplotype inference of polyploid genomes is feasible, is becoming feasible, or need new data types/computing methods to become feasible.

10. Diploid de novo assembly consists of two separate problems: assemble the complete haploid sequence (end-to-end assembly) of a chromosome, and assemble the diploid sequences of parental chromosomes. These two problems are related but have different challenges. The author should discuss the challenges and recent advances towards addressing these two problems separately. It will also be useful to comment on the preferred input data type for each algorithm/bioinformatic method mentioned in the text.

11. For polyploid haplotype assembly, I cannot get a clear sense about whether we are close to, or how far we are from being able to achieve polyploid assembly. Given the focus of this review on whole-chromosome scale haplotype analysis, the author should describe start-of-the-art approaches in the context of whole-chromosome scale analysis.

12. In the section on metagenome assembly, the author "presented" a general workflow in Fig. 4b. Is this a description of others' work, or does this reflect the author's new perspective?

13. I enjoyed reading the author's perspectives about remaining challenges but hope the author can comment on what computational innovations, new sequencing data types, or some combinations of both may help address these challenges.

Figures:

1. In Figure 1, it isn't clear what distinguishes between "short-range" or "long-range" sequencing based on the schematic drawing. The representation of Hi-C contacts is misleading as the insert does not span the intermediate DNA segments and Hi-C contacts are not always intra-chromosomal (in cis).

2. In Figure 2, the difference between local haplotype phasing and chromosome-scale haplotype phasing in the top panel isn't obvious. The schematic model of trio haplotype phasing is also not straightforward to understand, neither is polyploid haplotype phasing.

3. In Figure 3, the utility of long-range sequencing isn't clearly represented as they appear as short reads. It is also unclear what is the distinction between hybrid uncollapsed assembly and semi-collapsed assembly (hybrid approach). Do these two approaches represent different implementations of "Collapsed assembly"?

**Reviewer 2**

**Comments to author:**

This manuscript reviews the current state and challenges in reconstruction of full-chromosome haplotypes. The author briefly discusses the recent advances in genome sequencing technologies with the main focus on computational approaches for reference-based and de novo phasing of diploid, polypoid and metagenomic samples. This review concludes with describing the future challenges.

I see several major deficiencies in this review.

1. The proper context of review topic is missing. After reading the introduction I was not convinced that chromosome-scale haplotyping is a real bottleneck in the current genomics research. The papers that are referenced in the introduction mainly describe the use of local rather than chromosome-scale haplotype information. Some of the references do not seem to 'belong' to the scope of the review. A good example is reference #9, where study deals with phase separation - not in the sense of haplotypes, but liquid-liquid phase separation in the cells.

2. The deeper focus on algorithms and relatively narrow description of application context, technology developments and future prospects makes this review less appealing to a general reader. In its current form, the manuscript would be better suited for a specialized journal in the field of bioinformatics.

3. Manuscript is difficult to read, it would benefit from editing for style and clarity. While reading I constantly had a feeling that it was written in a hurry:

a)	some sentences were not thought through, e.g. this one from introduction: "Traditionally, association to diseases with only simple variations of genetic variation, such as SNPs, are studied with respect to a linear reference sequence, , for example, two SNPs, rs9494885 and rs2230926 in the TNFAIP3 gene, have known correlation with scleritis disease5"

b)	Mismatch between subsection headers and content. For example, "Short-range sequence information" discusses long-read technologies: PacBio, ONT, 10x genomics and BioNano "… that can fingerprint megabase segments …". It is a good idea to use more specific headers, in this case "short-range haplotype information"

c)	Readers will be learning new concepts from review - it is important to give proper definitions. E.g.: "Haplotypes are combinations of alleles or SNPs from multiple genetic loci on the same chromosome". This is a misleading definition - SNPs stands for single nucleotide polymorphisms, while haplotypes consist of alleles. Another example is definition of haplotype block - "a genomic regions of the chromosomes that are phased together in a segments" - I am not sure what author was trying to say here.

d)	Some terms are present in glossary, but not in the review, e.g. "allele-specific expression", "compound heterozygosity" or "epistatic interactions"

e)	Literature references - use reference manager and stick to uniform referencing format, some citations contain incomplete information (5,8, 40, 48, 59 and others) which makes them difficult or impossible to find.

f)	A good review typically features nice figures and schemes that help to grasp basic concepts and ideas. The illustrations that come with this review are very basic, same-type, fast-made from lots of horizontal lines (reads) and text.

Minor points:

Change "we" into "I" throughout the text, this is single author paper.

What is "local sequencing technologies", page 6?

"reads that can span between a few kilobases…", better written as "reads representing genomic segments of a few kilobases", page 6

**Authors Response**

**Point-by-point responses to the reviewers’ comments:**

Reviewer reports:

Reviewer #1: In this review, Shilpa Garg summarized recent progress towards whole-chromosome scale haplotype reconstruction and assembly, focusing on computational approaches, and then discussed applications and limitations of these methods. Although there has been some excellent reviews on haplotype-resolved genomic analysis, e.g., Snyder, Adey et al., Nature Review Genetics 2015, the topic of whole-chromosome haplotype analysis that is the focus of the current review should benefit the general audience.

While I enjoy reading the review, I feel the following areas can be improved with some additional discussion/clarification.

Comments:

1. The motivation for deriving whole-chromosome haplotype knowledge and its immediate applications weren't clear based on the Introduction. The various applications listed by the author, including complex genomic variation, variants in regulatory elements and allele-specific gene expression, and evolutionary biology, have been largely restricted to locus-specific analysis but not reaching the whole-chromosome scale. It is unclear how whole-chromosome haplotype analysis may transform these areas of research or what new questions will be enabled by whole-chromosome haplotype knowledge. On a minor point, Ref.11 was not related to cancer genome analysis even though the approaches developed in this study may be applicable. The immediate applications of whole-chromosome haplotype knowledge in cancer genome analysis is also not obvious given that the prevalence of allelic imbalance in aneuploid genomes can enable whole-chromosome scale haplotype phasing without using any of the recent methods.

*Thank you for your comment. Good point. I now included examples of compound heterozygosity and keratitis-ichthyosis-deafness syndrome that commonly occur due to interactions between mutations on the whole-genome scale. Additionally, I included phenomena of extrachromosomal DNA and aneuploidy centromeric context in cancer genomes that require recent whole-chromosome scale haplotype approaches to identify and characterize these events at the haplotype base resolution.*

2. The author discussed both technological advances and computational innovations that are necessary to make whole-chromosome scale haplotype reconstruction feasible. For technological advances, the author mentioned "short-range sequence information" from "long-read" sequencing; this may confuse the general audience who will associate "short-range sequence information" with illumina short-read sequencing. It will be useful to define "short-range" sequence information and explain why "long-read" sequencing only gives "short-range" sequence information and but not "long-range" sequence information. All the technologies mentioned by the author, including PacBio SMRT, 10X genomics, and BioNano optical mapping, are commonly referred as "long-range" sequencing.

*Yes, I agree with your point. I defined both short-range and long-range at the beginning of respective paragraphs in the revised version. For example, “In the era of third-generation sequencing technologies, I define short-range sequencing that produces genomic fragments (reads) spanning up to several hundreds of kilobases or megabases of the genome, but can’t connect across tens of megabase sized regions on the whole-genome scale. Long-range sequencing consists of technologies that produce genomic fragments spanning across centromeres, thereby providing information to connect p and q arms over the entire genome, and connecting across multi-megabases regions”.*

3. For long-range sequence information, the author mentioned both Hi-C and Strand-Seq. Whereas Hi-C can be implemented both on bulk samples and in single cells, Strand-Seq strictly requires cycling single cells. Both technologies provide long-range sequence information, but their utilities are differentiated by the experimental procedure. The author should discuss the sample requirements of these technologies and their implications for sequencing applications. It is also useful to discuss similar requirements for sequencing technologies that the author listed under "short-range" sequence information.

*I understand your point. To explain the sample requirements and DNA prep well, I require at least a few paragraphs for each technology. Considering the computational focus of this manuscript and word limit, I referenced papers explaining sample requirements, protocols for each technology in the revised manuscript.*

4. The section on Reference-based haplotype reconstruction includes both statistical phasing based on "reference haplotype data" from large cohorts, and molecular haplotyping that is based on long reads aligned to the reference genome. The section title causes confusion between these two applications. I also found the author's discussion of statistical phasing to be quite superficial. Perhaps the author can simply point to the original studies (Ref.33-36) and briefly describe why statistical phasing cannot reach the whole-chromosome scale.

*I agree. Text has been updated in the revised manuscript. “Traditionally, reference panels of more than 100,000 individuals (large-scale projects such as UK10K) are genotyped and used to assign, probabilistically, the most likely local phase of the target sample based on the underlying evolutionary model. This statistical phasing technique limits chromosome-scale haplotypes production because the ancestry tracts from populations or the Mendelian laws of inheritance from trios has only local information to produce the haplotypes. Phasing directly from sequencing reads, that is, the direct observation of two or more variants on a single molecule or in paired reads derived from the same molecule, overcomes the above limitations. The process of obtaining haplotypes directly from long-read and chromosome-scale sequencing data of a single individual — as opposed to phasing from genotypes by population inference or genetic analysis of pedigrees — is known as molecular haplotyping.”*

5. In the section titled "diploid phasing", the author listed several methods including WhatsHap, HapCut2, and ProbHap. These methods are applicable to different types of sequencing data and can generate haplotype inference with different completeness and accuracy depending on the input data. Given the focus on computational methods, the author should discuss these differences and how these methods are optimized for different input data.

*Manuscript updated suitably: “The performance of these methods is comparable and are optimized for different input data types, for example, all three methods produce comparable phasing completeness/accuracy/continuity using short-range sequencing, while HapCut2 and WhatsHap produce one large haplotype block on the chromosome-level using a combination of short-range and long-range sequencing data types.”*

6. I like the author's description of the comptuational algorithms of diploid phasing. However, the author's statement that "HapCut2 can typically phase only 50% of variants (Ref.42)" is misleading. I think the author was referring to HapCut2 can only phase 50% of variants using Hi-C data of 40x (Edge et al. claimed that HapCut2 can phase 60-70% of all variants). It is necessary to describe the input data for the quoted benchmark and comment on whether this can be improved with more data.

*Thank you for your comment. Yes, I included the technology and suggested an improvement using uniform coverage data. The challenge is to produce such data in the labs. Thus, hybrid approaches are the current norm for complete and accurate chromosome-scale phasing.*

7. For Hybrid approaches, the author should describe what input data are required to achieve accurate whole-chromosome haplotype inference (hammming error < 1% and switch error rate < 0.5%), including the type of long-range sequencing data, depth requirement, variants etc. The examples of phased Ashkenazi and Chinese human genomes are not convincing as "impressive advances in the production of high-quality chromosome-scale phasing," as these genomes had been phased by trio sequencing and are often used for optimizing the computational methods. In addition, the author should cite the study by Bansal, Bioinformatics 2019, which combines reference-based phasing and Hi-C haplotyping to achieve whole-chromosome scale haplotype inference. The utility of this method appears to be broader than the others as it only uses one data type.

*Done*

8. For single-cell phasing, the first paper that demonstrated its utility is Ref.37 but that was not cited appropriately.

*Done*

9. The author gave a nice introduction on polyploid phasing but I am not sure whether whole-chromosome haplotype inference of polyploid genomes is feasible, is becoming feasible, or need new data types/computing methods to become feasible.

*This has been explained in the revised version. New specific algorithms are required to be designed to produce chromosome-scale haplotypes. “While these methods represent an important step forward in polyploid phasing, hybrid methods that leverage HiFi, or alternatively linked-read data, and long-range Hi-C data can potentially produce chromosome-scale haplotypes for complex repetitive polyploids. In the near future, further algorithmic developments in local k-mer strategies and graphs-based approaches that focus on every haplotype could enable chromosome-scale phasing in large polyploid genomes.”.*

10. Diploid de novo assembly consists of two separate problems: assemble the complete haploid sequenc e (end-to-end assembly) of a chromosome, and assemble the diploid sequences of parental chromosomes. These two problems are related but have different challenges. The author should discuss the challenges and recent advances towards addressing these two problems separately. It will also be useful to comment on the preferred input data type for each algorithm/bioinformatic method mentioned in the text.

*This is clarified in the revised version. For example: “In de novo haplotype assembly, there are two major related challenges: finding ordering of sequencing reads and distinguishing reads to haplotypes. To find ordering of reads, the brute-force approach is to align all reads to all other reads, where the performance is directly proportional to square of the number of reads. In repetitive regions, finding alignments of reads is even more expensive. For systematic study, overlap-based or de Bruijn graph based techniques are used. To solve another challenge of finding haplotype of reads, the commonly used approach was heterozygous SNPs to partition reads to haplotypes in the space of single consensus sequence (due to high error rates in long-read PacBio and ONT data), however, with latest advancements in Hifi, allowed to separate reads to haplotypes during the overlapping step as discussed below.”*

11. For polyploid haplotype assembly, I cannot get a clear sense about whether we are close to, or how far we are from being able to achieve polyploid assembly. Given the focus of this review on whole-chromosome scale haplotype analysis, the author should describe start-of-the-art approaches in the context of whole-chromosome scale analysis.

*Very good point. I now updated the content to focus the polyploid assembly on the whole-chromosome scale. I added that the haplotype construction for genomes with chromosome sizes of tens of megabases in size can be done in the near future, whereas more sophisticated algorithm development is required for large complex genomes. The algorithms that leverage information from HiFi and Hi-C sequencing to separate haplotypes during the assembly process could be helpful for constructing polyploid haplotype assembly on the whole-chromosome level.*

12. In the section on metagenome assembly, the author "presented" a general workflow in Fig. 4b. Is this a description of others' work, or does this reflect the author's new perspective?

*Fig. 4b reflects the author's perspective.*

13. I enjoyed reading the author's perspectives about remaining challenges but hope the author can comment on what computational innovations, new sequencing data types, or some combinations of both may help address these challenges.

*Good point. Manuscript updated suitably. “Further increase in read lengths to several megabases in size, and/or reads with spatial coordinates, and/or longer reads with >99% accuracy, as well as innovations in k-mer and graph-based strategies to distinguish variations/motifs may enable exploration of high-resolution haplotypes in these human centromeric regions. ”*

Figures:

1. In Figure 1, it isn't clear what distinguishes between "short-range" or "long-range" sequencing based on the schematic drawing. The representation of Hi-C contacts is misleading as the insert does not span the intermediate DNA segments and Hi-C contacts are not always intra-chromosomal (in cis).

*We improved the visual representation to distinguish between short-range and long-range. We additionally show that the Hi-C data is sparse compared to long-read datasets and contain long-range information. It seems that the figure will become too busy to show inter/intra-chromosomal interactions in Hi-C, so we restrict the representation to only one chromosome.*

2. In Figure 2, the difference between local haplotype phasing and chromosome-scale haplotype phasing in the top panel isn't obvious. The schematic model of trio haplotype phasing is also not straightforward to understand, neither is polyploid haplotype phasing.

*Thank you for your observation. We have improved the figure to make it visually appealing and improved the presentation of chromosome-scale haplotype phasing. Due to space limitation in the figure, we removed the trio haplotype phasing, as it can be clear from the text directly. The trio haplotype phasing has limited applications due to difficulty to acquire families and three times sequencing data than for a single individual.*

3. In Figure 3, the utility of long-range sequencing isn't clearly represented as they appear as short reads. It is also unclear what is the distinction between hybrid uncollapsed assembly and semi-collapsed assembly (hybrid approach). Do these two approaches represent different implementations of "Collapsed assembly"?

*We have clarified in the figure by putting a box that represents the step of the collapsed haplotype process. As observed, in the semi-collapsed approach, the first step is collapsed haplotype based, while the rest of the steps are purely graph-based that retains the full haplotype information without any collapses. For example, Hi-C data steps are different in collapsed and semi-collapsed approaches. We also updated the insert sizes of Hi-C data (long-range sequencing).*

----

Reviewer #2: This manuscript reviews the current state and challenges in reconstruction of full-chromosome haplotypes. The author briefly discusses the recent advances in genome sequencing technologies with the main focus on computational approaches for reference-based and de novo phasing of diploid, polypoid and metagenomic samples. This review concludes with describing the future challenges.

I see several major deficiencies in this review.

1. The proper context of review topic is missing. After reading the introduction I was not convinced that chromosome-scale haplotyping is a real bottleneck in the current genomics research. The papers that are referenced in the introduction mainly describe the use of local rather than chromosome-scale haplotype information. Some of the references do not seem to 'belong' to the scope of the review. A good example is reference #9, where study deals with phase separation - not in the sense of haplotypes, but liquid-liquid phase separation in the cells.

*Done.*

2. The deeper focus on algorithms and relatively narrow description of application context, technology developments and future prospects makes this review less appealing to a general reader. In its current form, the manuscript would be better suited for a specialized journal in the field of bioinformatics.

*In the updated manuscript, an attempt has been made to make it more appealing to a general reader.*

3. Manuscript is difficult to read, it would benefit from editing for style and clarity. While reading I constantly had a feeling that it was written in a hurry:

*Done.*

a) some sentences were not thought through, e.g. this one from introduction: "Traditionally, association to diseases with only simple variations of genetic variation, such as SNPs, are studied with respect to a linear reference sequence, , for example, two SNPs, rs9494885 and rs2230926 in the TNFAIP3 gene, have known correlation with scleritis disease5"

*Done*

b) Mismatch between subsection headers and content. For example, "Short-range sequence information" discusses long-read technologies: PacBio, ONT, 10x genomics and BioNano "… that can fingerprint megabase segments …". It is a good idea to use more specific headers, in this case "short-range haplotype information"

*It’s both sequence and haplotype information, we updated it.*

c) Readers will be learning new concepts from review - it is important to give proper definitions. E.g.: "Haplotypes are combinations of alleles or SNPs from multiple genetic loci on the same chromosome". This is a misleading definition - SNPs stands for single nucleotide polymorphisms, while haplotypes consist of alleles. Another example is definition of haplotype block - "a genomic regions of the chromosomes that are phased together in a segments" - I am not sure what author was trying to say here.

*Done*

d) Some terms are present in glossary, but not in the review, e.g. "allele-specific expression", "compound heterozygosity" or "epistatic interactions"

*Done*

e) Literature references - use reference manager and stick to uniform referencing format, some citations contain incomplete information (5,8, 40, 48, 59 and others) which makes them difficult or impossible to find.

*Done*

f) A good review typically features nice figures and schemes that help to grasp basic concepts and ideas. The illustrations that come with this review are very basic, same-type, fast-made from lots of horizontal lines (reads) and text.

*The Figures have been updated and clarified as discussed above*

Minor points:

Change "we" into "I" throughout the text, this is single author paper.

What is "local sequencing technologies", page 6?

*Done*

"reads that can span between a few kilobases…", better written as "reads representing genomic segments of a few kilobases", page 6

*Done*

**Second round of review**

**Reviewer 1**

I will only comment on where the author's revision does not fully address my critique in the first review.

1. About the relevance of whole-chromosome haplotype inference. I am not convinced that the examples given the authors indeed require whole-chromosome haplotype knowledge. For example, the advantage of whole-chromosome haplotype knowledge than local haplotype knowledge for the analysis of ecDNA or aneuploidy in cancer genomes has not been well demonstrated. I suggest the author discuss what questions CAN BE addressed with whole-chromosome haplotype, instead of discussing what applications are already available. Above all, whole-chromosome haplotype inference is only available very recently and its potential applications are not fully developed.

Related to 3,5,7: Because Hi-C and Strand-Seq are very different data types and require different computational strategies, the author should treat them as separate data types with different companion computational methods. For example, I am not sure WhatsHap can work on Hi-C data or HapCut2 can work on Strand-Seq data. Given the focus of the review is on computational methods, the author should discuss how these different algorithms tackle the problem of long-range haplotype inference differently to take advantage of the different features of input data.

**Reviewer 2**

The author has improved the manuscript and addressed my points satisfactory.

**Authors Response**

**Point-by-point responses to the reviewers’ comments:**

Reviewer #1: I will only comment on where the author's revision does not fully address my critique in the first review.

1. About the relevance of whole-chromosome haplotype inference. I am not convinced that the examples given the authors indeed require whole-chromosome haplotype knowledge. For example, the advantage of whole-chromosome haplotype knowledge than local haplotype knowledge for the analysis of ecDNA or aneuploidy in cancer genomes has not been well demonstrated. I suggest the author discuss what questions CAN BE addressed with whole-chromosome haplotype, instead of discussing what applications are already available. Above all, whole-chromosome haplotype inference is only available very recently and its potential applications are not fully developed.

*The manuscript updated suitably: “The inference of whole-chromosome haplotypes have clinical relevance: having both variants on the same allele (cis) lead to a specific ( for example, super-responder) phenotype; when those variants were on separate alleles (trans) do not”*

Related to 3,5,7: Because Hi-C and Strand-Seq are very different data types and require different computational strategies, the author should treat them as separate data types with different companion computational methods. For example, I am not sure WhatsHap can work on Hi-C data or HapCut2 can work on Strand-Seq data. Given the focus of the review is on computational methods, the author should discuss how these different algorithms tackle the problem of long-range haplotype inference differently to take advantage of the different features of input data.

*The manuscript updated suitably: “State-of-the-art hybrid phasing tools are WhatsHap and HapCut2. HapCut2 directly works on reads from long-read and Hi-C sequencing using likelihood inference optimization, while WhatsHap operates on Strand-seq haplotypes and long-read reads using MEC formulation. ”*
